# Artificial intelligence in aortic CT angiography: current applications and future perspectives

**DOI:** 10.3389/fcvm.2025.1674486

**Published:** 2026-01-14

**Authors:** Jingkai Xu, Jinjin Liu, Guoquan Cao

**Affiliations:** 1 The First School of Medicine, School of Information and Engineering, Wenzhou Medical University, Wenzhou, China; 2 Department of Radiology, The First Affiliated Hospital of Wenzhou Medical University, Wenzhou, China

**Keywords:** aortic diseases, artificial intelligence, computed tomography angiography, convolutional neural network, deep learning

## Abstract

Artificial intelligence (AI) is revolutionizing cardiovascular imaging, with aortic computed tomography angiography (CTA) emerging as a prominent area of application. CTA imaging is essential for the diagnosis, risk stratification, and treatment planning of aortic diseases. However, conventional CTA techniques face limitations such as radiation exposure, contrast agent risks, and reliance on manual interpretation. The integration of AI into aortic CTA offers innovative solutions across multiple domains. AI can enhance image quality, automate anatomical segmentation, improve diagnostic accuracy for aortic emergencies, and provide quantitative tools for prognostic evaluation following interventions like endovascular aortic repair. Furthermore, this review provides the analysis of emerging techniques, including advanced image synthesis methods, Vision Transformer architectures, multi-task learning, weakly supervised learning, and the paradigm shift introduced by Foundation Models, emphasizing their potential for clinical application. This work comprehensively summarizes the current applications and nascent technological paradigms of AI in aortic CTA, along with existing challenges and future research directions.

## 1 Introduction

Aortic diseases are significant large-vessel pathologies that represent a substantial threat to human health. These conditions encompass aortic dissection (AD), aneurysm, coarctation, and functional abnormalities such as reduced elasticity or impaired distensibility of the aortic wall (1). Among them, AD stands out due to its acute onset, complex presentation, and high fatality rate if not promptly diagnosed and treated. The incidence of AD is estimated to be 5–30 cases per million people per year, with men more commonly affected, as reported by 2022 American College of Cardiology (ACC)/American Heart Association (AHA) Aortic Disease Guideline (2). Acute presentations of aortic diseases highlight the critical need for early detection and precise risk assessment. Given their unpredictable course and severe complications, accurate diagnosis and timely management of aortic diseases are critical to improving patient outcomes. Computed tomography angiography (CTA) currently serves as the primary imaging modality for evaluating the aorta and its branches, owing to its high efficiency and diagnostic accuracy. It is extensively employed for disease diagnosis and postoperative surveillance (3). Nevertheless, traditional CTA presents several limitations. The use of iodinated contrast agents may cause adverse reactions, with an overall prevalence of hypersensitivity reactions of approximately 0.73% (4). Additionally, the manual or semi-automatic segmentation of CTA images is highly time-consuming, substantially reducing clinical efficiency; for example, delineating a single Standford type B AD case may require approximately 1–1.5 h of expert annotation (5). Furthermore, the diagnostic accuracy often relies heavily on the radiologist's experience, introducing potential for subjective bias and inter-observer variability.

In recent years, artificial intelligence (AI) has emerged as a powerful adjunctive tool in medical imaging. AI, a subfield of computer science, seeks to emulate and augment human cognition through computational methodologies (6, 7). Traditional machine learning—encompassing supervised, unsupervised, and reinforcement learning—has formed the conceptual foundation of AI in imaging. Supervised learning remains predominant in medical imaging due to the availability of well-annotated datasets and the interpretability of label-driven outputs, which align with clinical validation requirements. It enables models to learn mappings from input data to known outcomes, supporting tasks such as classification and regression (8–10). In contrast, unsupervised learning operates on unlabeled data, seeking to uncover latent patterns or intrinsic data structures, with common techniques include clustering and dimensionality reduction (11). Reinforcement learning by enabling agents to learn optimal behaviors through interaction with an environment and receiving feedback in the form of rewards or penalties (12).

Deep learning (DL) has become the dominant paradigm in medical imaging AI. Convolutional neural networks (CNNs) and their variants, such as fully convolutional networks (13) and U-Net (14), have achieved remarkable success in image reconstruction, segmentation, and diagnosis prediction. In recent years, transformer-based architectures, originally developed for natural language processing, have been successfully adapted to visual domains, providing enhanced capability to capture long-range dependencies and global contextual relationships within images. Vision Transformers (ViTs) and hybrid CNN–Transformer models have demonstrated robust performance across diverse imaging modalities (15, 16). Concurrently, foundation models—large-scale, pre-trained networks—have redefined model development by enabling efficient pre-training on massive heterogeneous datasets followed by fine-tuning for domain-specific tasks. Meanwhile, diffusion models have emerged as state-of-the-art generative frameworks capable of synthesizing anatomically realistic medical images, supporting data augmentation, cross-modality translation, and artifact reduction (17).

**Figure 1:**
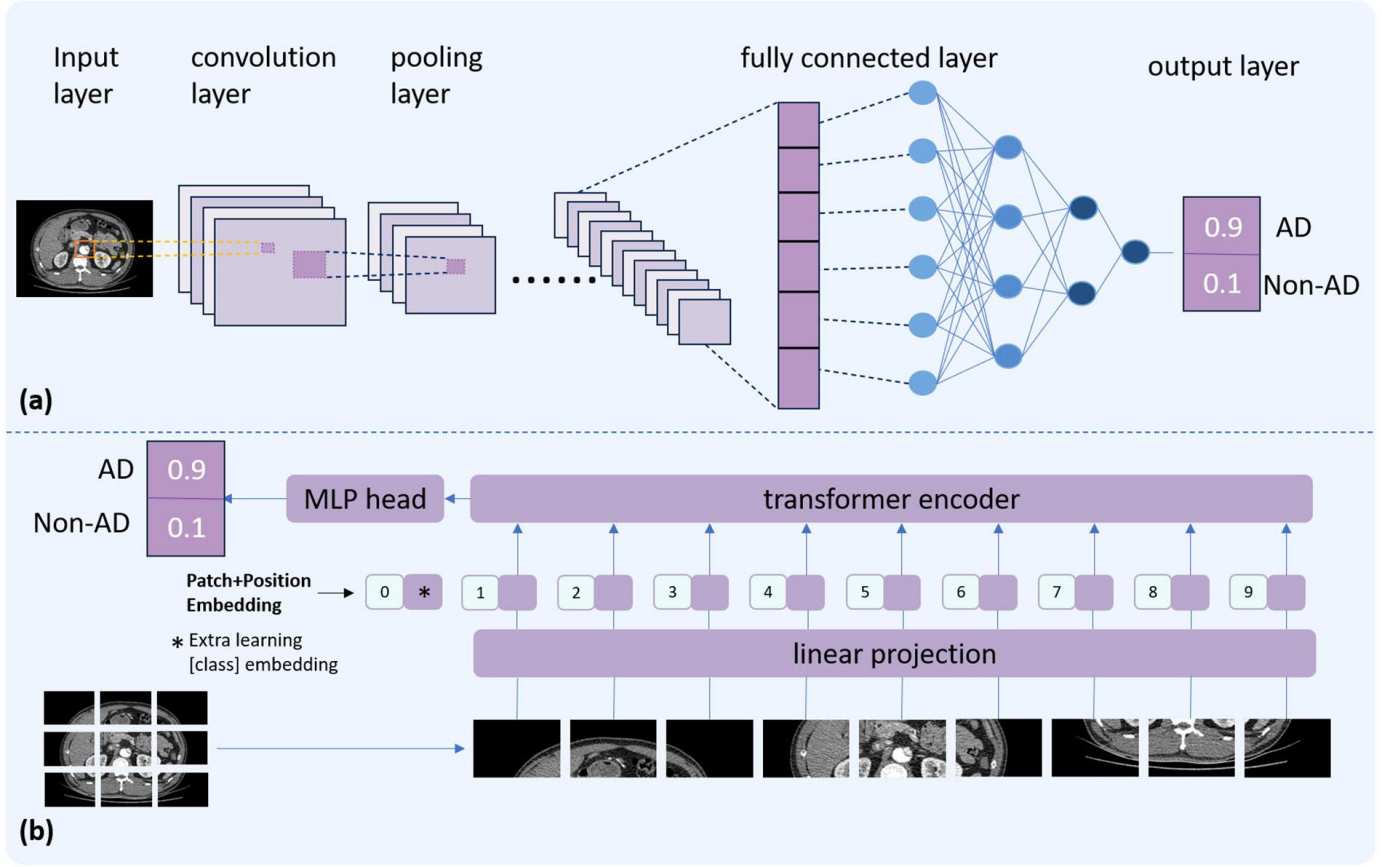
Comparative illustration of two deep learning architectures. The top panel **(a)** depicts the basic structure of a CNN. The bottom panel **(b)** presents the ViT architecture.

Figure 1 illustrates the basic architectures of a CNN and a ViTs. A typical CNN consists of several key components: an input layer to receive raw data, convolutional layers that apply learnable filters (commonly 3 × 3 or 5 × 5 kernels) to extract local features, pooling layers that reduce spatial dimensionality to minimize computational complexity and mitigate overfitting, and fully connected layers that map the extracted features to the output space, yielding final predictions. Instead of processing images through local convolutional filters, ViTs divides an image into a sequence of fixed-size patches (commonly16 × 16 pixels), flattens each patch into a vector, and projects it into an embedding space analogous to word tokens in a sentence. Positional encodings are added to preserve spatial information. These patch embeddings are then processed by a series of Transformer encoders based on self-attention mechanisms. Finally, the final result is generated through a multi-layer perceptron (MLP) classification head.

AI technologies have demonstrated remarkable capabilities in enhancing medical imaging analysis, particularly within CT-based modalities. In lung cancer screening, deep learning models have significantly improved the detection, classification, and prognosis prediction of pulmonary nodules on CT scans (18). Similarly, in coronary artery disease assessment, deep learning–based analysis of coronary CTA reduced image reconstruction time by 85% and diagnostic time by 80%, compared to traditional methods (19). Moreover, for acute neurological emergencies, explainable AI (XAI) frameworks have enabled precise localization of cerebral hemorrhages on CT, providing visual heatmaps to guide urgent interventions (20). These breakthroughs have paved the way for exploring AI's role in aortic imaging. By enhancing image quality, enabling automated segmentation, assisting diagnosis, and supporting risk stratification, AI holds the promise to augment CTA workflows and improve clinical decision-making for aortic diseases.

Numerous review articles have previously summarized the application of AI in cardiovascular imaging. However, their scope and specific focus exhibit considerable variation. Specifically, Yang (21) and Joshi et al. (22) primarily concentrated on applications related to coronary arteries, while Mastrodicasa et al. (23) and Asif et al. (24) addressed AD. Furthermore, the rapid and continuous evolution of AI technology suggests that earlier reviews may no longer adequately encompass the most recent advancements in this field. Therefore, the primary objective of this review is not to conduct another comprehensive reappraisal based on existing literature. Instead, after a concise overview of AI applications in aortic CTA, we proceed to delve into the exploration of cutting-edge technologies, including image synthesis, multi-task and weakly supervised learning, ViTs and foundation models. Furthermore, this review aims to provide critical insights into future research directions and clinical translation. Through this work, we strive to bridge the gap between technological advancements and clinical utility, fostering a deeper understanding of how AI is poised to reshape the landscape of aortic disease diagnosis and management.

## 2 AI applications in aortic CTA

### 2.1 Application of AI in image reconstruction

Image reconstruction algorithms play a pivotal role in determining the quality of CT imaging and the reliability of subsequent diagnostic interpretations. Among recent advances, AI, particularly deep learning reconstruction (DLR), has emerged as a novel approach that differs fundamentally from traditional reconstruction methods such as filtered back-projection (FBP) and iterative reconstruction (IR) methods.

DLR techniques can be broadly classified into two categories: direct DLR and indirect DLR (25). Direct DLR employs deep neural networks to directly transform raw projection data into high-fidelity reconstructed images. This end-to-end framework enables comprehensive optimization of the reconstruction process and enhances artifact and noise suppression. In contrast, indirect DLR applies DL models to images reconstructed via traditional algorithms such as FBP or IR. This approach focuses on post-processing to further reduce noise, enhance image quality, and suppress artifacts.

**Figure 2:**
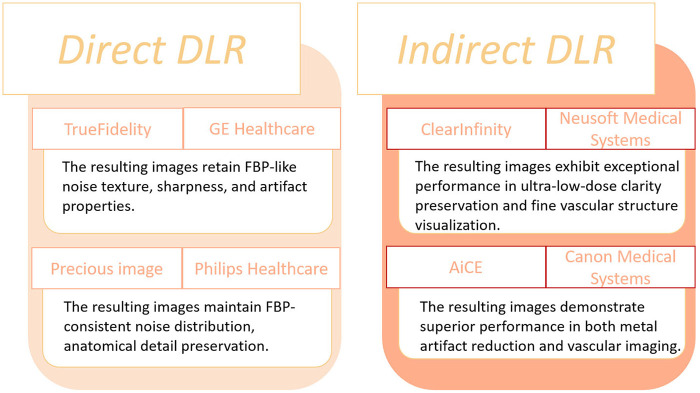
Currently commercially available DLR algorithms.

DLR offers several clinical advantages, including improved signal-to-noise ratio (SNR) and contrast-to-noise ratio (CNR), thereby facilitating lower radiation exposure and reduced contrast agent usage while maintaining diagnostic image quality. These capabilities exemplify the precise and effective integration of AI technologies within medical imaging workflows. Commercially available DLR algorithms are summarized in Figure 2 (25).

Subsequent innovations rapidly translated these ideas into clinical practice. Commercial engines such as AiCE (Canon Medical), TrueFidelity (GE Healthcare), and ClearInfinity (Neusoft Medical) integrated deep neural networks into standard CT workflows. For example, Cai et al. (26) demonstrated that combining AiCE with Single-Energy Metal Artifact Reduction (SEMAR) reduced metallic artifacts by nearly 47% while improving SNR and CNR. Similarly, Ding et al. (27) introduced a DLR-based “dark-blood” technique that enhanced vessel wall visualization in large-vessel vasculitis. Kojima et al. (28) further validated the clinical feasibility of low-tube-voltage CTA reconstructed with AiCE, achieving comparable quality to model-based IR while cutting reconstruction time by 83%. These studies collectively illustrated how DLR could simultaneously reduce radiation and reconstruction latency—two persistent clinical bottlenecks. Parallel developments by other vendors extended this trend. Using TrueFidelity, Heinrich et al. (29) demonstrated consistent noise reduction (up to 57%) and significant CNR improvement (44%–125%) across aortic segments. Li et al. (30) showed that pairing 70-kVp protocols with DLR achieved over 50% reductions in radiation and contrast dose while improving coronary CNR by 66%. Later, Kawai et al. (31) and Qi et al. (32) pushed the limits toward ultra-low-dose and low-contrast protocols, employing U-Net-based multi-scale architectures to maintain diagnostic quality even at 60 kVp. These technical refinements progressively transformed DLR from a research concept into a clinically viable solution for dose-conscious aortic CTA. The Applications that have been proposed and validated are summarized in Table 1.

**Table 1:** Clinical applications on DLR algorithms.

Authors	Year	DLR name	No. of patients	Main finding
Heinrich et al. (29)	2021	TrueFidelity	51	Compared with ASIR-V, DLR enables radiation dose reduction and significantly improves aortic CTA image quality, with and CNR increases up to 58% and 56%, and higher subjective ratings.
Li et al. (30)	2022	TrueFidelity	100	Compared with ASIR-V, DLR at reduced radiation dose by 54.5%, contrast dose by 50.6%.
Cai et al. (26)	2024	AiCE	47	The combination of AiCE and SEMAR significantly reduces noise and artifacts and enhances detection of endoleaks and thrombi after EVAR.
Ding et al. (27)	2024	AiCE	50	Compared with HIR, DLR in LVV patients improved overall image quality and vessel wall visualization, especially when combined with the “dark blood” technique.
Kojima et al. (28)	2024	AiCE	30	DLR images had lower radiation dose and contrast medium than those of HIR and MBIR.
Kawai et al. (31)	2024	TrueFidelity	34	Compared with HIR, DLR showed well-balanced arterial depictions and image quality.
Qi et al. (32)	2025	ClearInfinity	90	Compared with HIR, DLR allowed radiation dose reduction by 45% and contrast medium reduction by 50% in aortic CTA.

ASIR-V, adaptive statistical iterative reconstruction-V; CNR, contrast-to-noise ratio; CTA, computed tomography angiography; DLR, deep learning reconstruction; EVAR, endovascular aneurysm repair; HIR, hybrid iterative reconstruction; LVV, large vessel vasculitis; MBIR, model-based iterative reconstruction; SEMAR, single-energy metal artifact reduction; SNR, signal-to-noise ratio.

### 2.2 Application of AI in image segmentation

**Figure 3:**
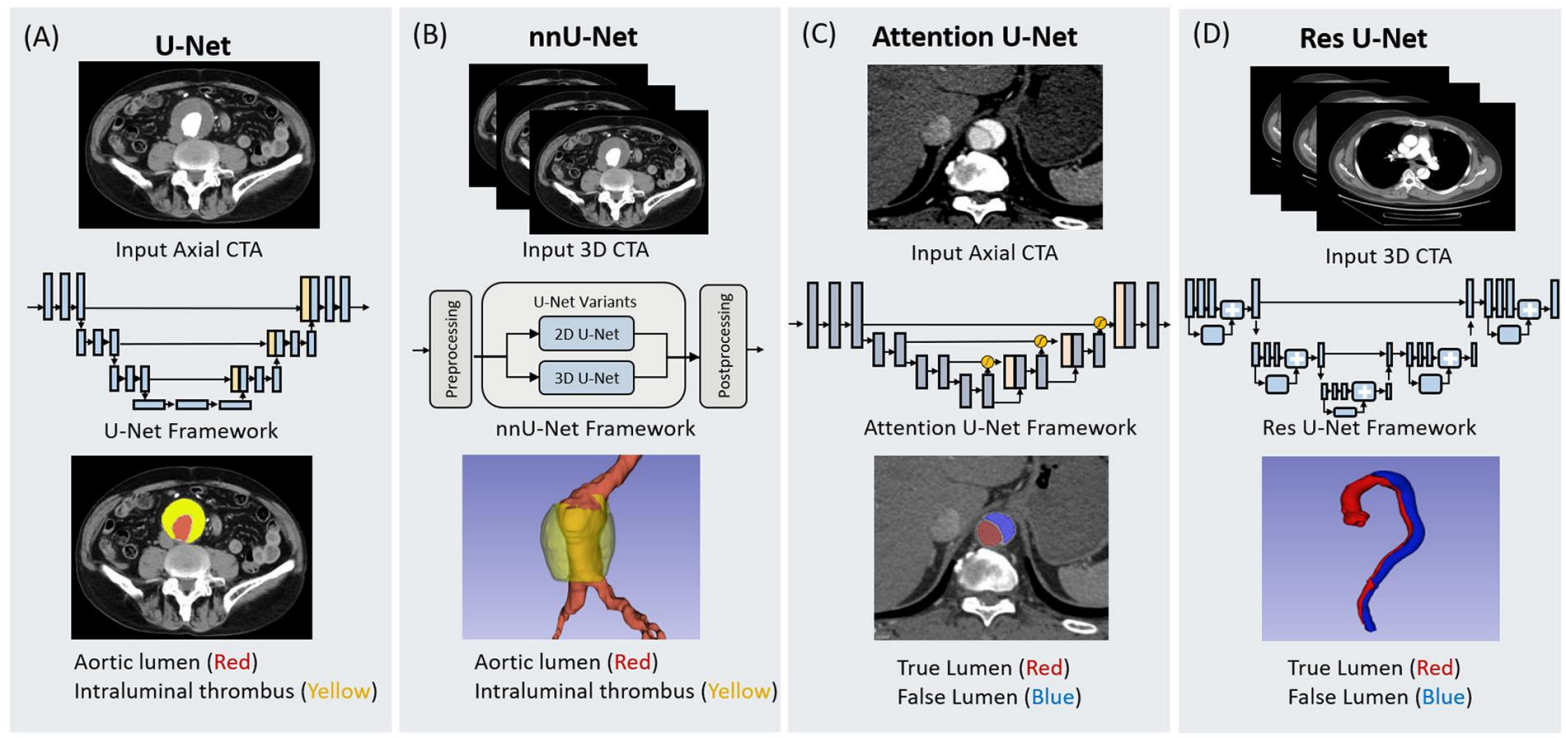
Schematic representation of four deep learning architectures utilized for aortic segmentation. The figure illustrates the input data modalities, network frameworks, and segmentation outputs for **(A)** U-Net, **(B)** nnU-Net, **(C)** Attention U-Net, and **(D)** Res U-Net. U-Net and nnU-Net are demonstrated in the context of abdominal aortic aneurysm segmentation, identifying the aortic lumen (red) and intraluminal thrombus (yellow) from 2D axial and 3D CTA inputs, respectively. Attention U-Net and Res U-Net are shown in the context of aortic dissection, distinguishing the true lumen (red) and false lumen (blue). The Attention U-Net employs attention gates to process axial slices, while the Res U-Net integrates residual learning blocks for 3D volumetric segmentation.

Accurate segmentation of the aorta and its associated pathological regions is critical prerequisite for automated analysis of aortic diseases. Effective segmentation delineates the anatomical boundaries of the aorta, providing a robust basis for subsequent tasks such as disease detection, morphological assessment, and treatment planning (33). Traditionally, aortic segmentation has relied primarily on manual segmentation or semi-automatic segmentation, both of which are time-consuming, labor-intensive, and susceptible to interobserver variability (34). With the rapid expansion of medical imaging data, traditional image segmentation methods are increasingly inadequate for meeting the demands of high-precision and fully automated segmentation (35, 36). Compared to conventional approaches, DL-based methods have demonstrated remarkable performance in medical image segmentation tasks. Currently, the majority of segmentation networks in clinical imaging are derived from the U-Net architecture. Figure 3 depicts the foundational U-Net framework alongside its advanced variants, demonstrating their respective architectures and workflows for aortic segmentation.

The transition from handcrafted to DL-based segmentation began with early efforts to quantify aortic aneurysms. Lareyre et al. (37) developed a fully automated pipeline combining level-set segmentation with adaptive thresholding, enabling 3D reconstruction of the lumen, thrombus, and calcification without manual intervention. Although this represented an important step toward automation, its reliance on handcrafted components limited generalizability. Building upon this, Brutti et al. (38) and Sieren et al. (39) introduced U-Net- and CNN-based architectures that achieved more robust and accurate abdominal aortic aneurysm (AAA) segmentation across diverse datasets, marking a clear improvement in reproducibility and efficiency. To further enhance comprehensiveness, Robbi et al. (40) integrated nnU-Net (41) and SegResNet (42) architectures in the BRAVE pipeline, enabling fully automated segmentation of multiple vascular and pathological structures—including lumen, thrombus, and calcified plaques—thus extending applicability to pre-endovascular aneurysm repair (EVAR) surgical planning. Together, these studies illustrate a methodological evolution from semi-automated to fully automated, multi-structure segmentation systems capable of handling the heterogeneity of aortic aneurysms.

Segmentation of AD introduced a new set of technical challenges, as complex vessel morphologies, overlapping lumens, and imaging artifacts often hindered accurate boundary delineation. Early CNN-based frameworks, such as the 3D model proposed by Yu et al. (43) for type B AD, achieved Dice similarity coefficient (DSC) exceeding 0.96 and demonstrated clinically usable measurements. However, the need for generalizable performance across subtypes motivated more sophisticated architectures. Guo et al. (44) incorporated both 2D and 3D components into an nnU-Net framework for type A AD, improving lumen and intimal flap segmentation, while Wobben et al. (45) enhanced boundary precision using residual symmetric U-Nets. These incremental improvements addressed local morphological complexity but still faced challenges in standardizing vessel geometry across patients. Consequently, Chen et al. (46) introduced a cascaded, multi-stage network with an aortic straightening module to enforce anatomical consistency, outperforming single-stage baselines. Driven by the clinical need for rapid, accurate postoperative analysis, subsequent studies explored more efficient and specialized solutions. Zhang et al. (47) combined contrast-enhanced segmentation and self-attention mechanisms for fine-grained lumen extraction in postoperative type B aortic dissection (TBAD), while Zhao et al. (48) employed a morphology-constrained mesh-based regression model that achieved a 94% DSC with sub-millimeter surface error in under a minute. Expanding to larger datasets, Lyu et al. (49) further refined the approach through a hybrid 2D–3D architecture balancing global and local feature extraction. Collectively, these advances trace a clear progression—from conventional encoder-decoder networks to hybrid, attention-based, and mesh-driven architectures—each addressing specific limitations of the previous generation and moving toward clinically deployable, precision-oriented segmentation of aortic dissection.

In summary, the development of DL-based segmentation for aortic CTA has progressed from basic rule-based tools to sophisticated, multi-network, attention-enhanced systems that directly respond to clinical needs for accuracy, speed, and reproducibility. This evolution demonstrates how technical innovation in network design has continually been driven—and validated—by practical challenges in vascular imaging. The effective methods proposed are selected and summarized in Table 2.

**Table 2:** Application of different models for aortic segmentation.

Authors	Year	Methods	No. of patients	Dataset sources	Annotation	Quality index	Quality index value
Lareyreet al. (37)	2019	Thresholding and Level-Set	40	Single center	Manual by an expert	DSC	0.93
						Sensitivity	0.90
						Specificity	0.99
Brutti et al. (38)	2022	Multi-view U-Net	85	Multi-center	Manual by a trained expert and by a trainee	DSC	0.89
Sieren et al. (39)	2022	CNN	191	Single center	Manual by two investigators	DSC	0.95
Robbi et al. (40)	2025	nnU-Net and SegResNet	20	Multi-center	N/A	DSC	0.88
Yu et al. (43)	2021	3D CNN	139	Single center	Manual by four experts	DSC	0.96
Guo et al. (44)	2024	nnU-Net	24	Single center	Manual by two experts	DSC	0.88/0.87
Wobben et al. (45)	2021	3D Res U-Net	147	Single center	Manual by a trained radiologist	DSC	0.85/0.84
Chen et al. (46)	2021	CNN	120	Multi-center	Manual by an expert	DSC	0.96/0.95
Zhang et al. (47)	2023	GLF-UNet	133	Multi-center	Manual by two experts	DSC	0.96/0.92/0.81
Zhao et al. (48)	2022	Morphology-Constrained Stepwise Deep Mesh Regression	35	Single center	Manual by two experts	DSC	0.94
						Sensitivity	0.93
Lyu et al. (49)	2021	CNN	42	Single center	Manual by an expert	DSC	0.92
						Sensitivity	0.91
						Precision	0.94

CNN, convolutional neural network; DSC, dice similarity coefficient; GLF, Global-Local Fusion.

### 2.3 Application of AI in the diagnosis of aortic diseases

The application of AI in the diagnosis of aortic diseases presents considerable potential, as timely and accurate diagnosis is critical for guiding treatment strategies and improving patient prognosis. Given the often complex clinical presentations and atypical imaging manifestations of aortic pathologies, rates of misdiagnosis and missed diagnosis have been reported to reach as high as 40% (50). Such diagnostic delays may lead to severe clinical consequences, including disease progression and increased mortality. AI-based technologies offer a potential solution to this challenge by enhancing diagnostic accuracy and efficiency. Early AI efforts focused on improving screening and detection. Spinella et al. (51) developed a 2.5D multi-view U-Net pipeline to segment the aortic lumen and thrombus in AAA screening. The model achieved high Dice scores (0.93 for lumen, 0.89 for thrombus) and provided fully automated processing, demonstrating how AI could streamline routine screening. Building on this concept of automated detection, Wada et al. (52) proposed a Deep convolutional neural network (DCNN)-based triage system capable of identifying aortic emergencies from cropped CTA images. By distinguishing emergency from control cases with high accuracy, this system highlighted the potential for AI to support rapid clinical triage.

The application of AI has also extended to non-contrast CT, addressing a key clinical need where contrast-enhanced imaging may be unavailable or contraindicated. Cheng et al. (53) introduced a threshold-based model utilizing volumetric differences between true and false lumens for AD detection, achieving high sensitivity and specificity. Hata et al. (54) applied an Xception-based architecture (55), showing robust performance independent of radiologist experience. Building on these approaches, Dong et al. (56) developed an attention-augmented You Only Look Once (YOLO) v5 model augmented with convolutional block attention module (CBAM), which outperformed conventional algorithms and human evaluations, illustrating how architectural innovations could enhance diagnostic precision in challenging imaging scenarios.

Beyond detection, AI has facilitated automated classification of aortic diseases. Huang et al. (57) implemented a two-step framework combining an Attention U-Net (58) with a 3D ResNeXt (59) network for Stanford classification of AD, achieving high specificity in distinguishing type A dissections. Complementing this, Laletin et al. (60) validated a two-stage CNN pipeline capable of both aortic segmentation and intimal flap detection, enabling rapid and accurate classification that directly supports clinical decision-making and triage.

AI has further advanced quantitative assessment of aortic morphology. Artzner et al. (61) demonstrated that automated thoracic aorta segmentation and diameter measurement achieved strong agreement with expert evaluations across both contrast and non-contrast CT. Pradella et al. (62) extended these capabilities with a fully automated, electrocardiogram-gated DL algorithm for guideline-compliant diameter measurement, showing concordance with radiologists for 87% of critical anatomical landmarks while markedly reducing measurement time. Nevertheless, discrepancies around the aortic root underscore the continued importance of anatomical verification in certain regions.

Collectively, these studies highlight the expanding role of AI in the diagnostic workflow of aortic diseases, automating quantitative assessment, and accelerating clinical decision-making. Looking forward, AI is poised to drive more intelligent and precise diagnostic and therapeutic strategies for aortic pathologies. The effective methods proposed are selected and summarized in Table 3.

**Table 3:** Application of different models in the diagnosis of aortic diseases.

Authors	Year	Methods	Research objectives	No. of patients	Dataset sources	Annotation	Quality index	Quality index value
Spinella et al. (51)	2023	2.5D CNN	Screening for AAA	73	Single center	Manual measurement by an expert	Accuracy	0.97
							Sensitivity	0.98
							Specificity	0.96
Wada et al. (52)	2023	DCNN	Screening for aortic emergencies	436	Single center	Manual by a radiologist	AUC	0.995
Cheng et al. (53)	2024	3D full-resolution U-Net	Screening for AD	320	Multi-center	Manual by radiologists	Accuracy	0.938
							Sensitivity	0.916
							Specificity	0.956
Hata et al. (54)	2021	Xception CNN	Screening for AD	170	Single center	Manual classification by an expert	AUC	0.940
							Accuracy	0.900
							Sensitivity	0.918
							Specificity	0.882
Dong et al. (56)	2024	YOLOv5-CBAM	Screening for AD	480	Multi-center	Bounding box labeling by two Radiologists	AUC	0.938
							Accuracy	0.915
							Sensitivity	0.900
							Specificity	0.929
Huang et al. (57)	2022	2-step hierarchical neural network	Stanford classification of AD	130	Single center	Labeled by two radiologists and one senior resident	Sensitivity	0.9545
							Specificity	0.9855
Laletin et al. (60)	2024	Two-stage CNN	Stanford classification of AD	1,303	Multi-center	Stanford classification by three radiologists	Sensitivity	0.942
							Specificity	0.973
Artzner et al. (61)	2022	DI2IN and Deep Reinforcement Learning	Measures of diameter	122	Single center	Manual measurement by two radiologists	ICC	≥0.961
Pradella et al. (62)	2021	DI2IN and Deep Reinforcement Learning	Diameter measurements of the thoracic aorta	405	Single center	semi-automatic measurement by three radiologists	Coherent results rate	87.0%

AAA, abdominal aortic aneurysm; AD, aortic dissection; AUC, area under the curve; CBAM, convolutional block attention module; CNN, convolutional neural network; DCNN, deep convolutional neural network; DI2IN, deep image-to-image network; ICC, intra-class correlation coefficient.; YOLOv5, you only look once version 5.

### 2.4 Application of AI in prognostic evaluation of aortic diseases

Prognostic assessment of aortic diseases is often hindered by subjectivity and limited predictive accuracy. AI techniques offer the ability to learn latent patterns from large-scale clinical and imaging datasets, thereby enabling objective evaluation of treatment outcomes, complication prediction, and risk stratification for patients. Recent studies have increasingly focused on the application of AI in prognostic modeling for aortic diseases, particularly in the context of post-EVAR.

Initial AI applications aimed to identify postoperative complications. Talebi et al. (63) developed a DL model to detect endoleaks following EVAR. Trained on CTA images from patients with and without endoleaks, the model achieved high accuracy and area under the curve (AUC), demonstrating the feasibility of AI-assisted complication detection.

Building on this, Olivier et al. (64) and Coatsaliou et al. (65) evaluated the AI software PRAEVAorta 2 for post-EVAR follow-up management. Their studies showed that the software could accurately quantify AAA sac volume changes over time, with strong concordance to semi-automatic methods, and enabled more sensitive detection of early sac progression. Importantly, PRAEVAorta 2 also identified endoleaks, including low-flow type II endoleaks that are frequently under-recognized in routine clinical assessments, demonstrating higher sensitivity and specificity compared to conventional radiology reports.

Beyond complication detection, AI has facilitated precise and automated measurement of aortic morphology. Wegner et al. (66) assessed Augmented Radiology for Vascular Aneurysm (ARVA), an AI tool for automatically measuring maximum aortic diameters in complex aneurysms pre- and post-EVAR. ARVA's measurements were comparable to those of expert clinicians, with small median absolute differences, confirming its accuracy for clinical application. Similarly, Adam et al. (67) validated ARVA for automatic determination of maximum aortic diameters on a series of CTA scans, achieving high agreement with manual measurements and demonstrating reliability for longitudinal monitoring.

Collectively, these studies demonstrate that the application of AI, in post-EVAR follow-up can significantly improve the sensitivity and efficiency of detecting complications such as endoleaks, and enable more accurate and automated assessment of aortic morphological changes. Compared to traditional manual interpretation, AI-based imaging analysis tools offer notable advantages by delivering rapid, quantitative, and standardized surveillance. With further large-scale and multicenter validation, these technologies hold the potential to support clinical decision-making, enhance the quality and efficiency of long-term follow-up, and enable accurate and non-invasive prognosis prediction for patients with aortic diseases. The effective methods proposed are selected and summarized in Table 4.

**Table 4:** Summary of studies on the application of AI in prognostic evaluation.

Authors	Year	Methods	Research objectives	No. of patients	Quality index	Quality index value
Talebi et al. (63)	2020	Endoleak Augmentor and U-Net	Detection of endoleaks	50	Accuracy	0.95
					AUC	0.99
Olivier et al. (64)	2025	PRAEVAorta 2	Measurements of aneurysm sac volume	49	ICC	0.94
Coatsaliou et al. (65)	2024	PRAEVAorta 2	Detection of endoleaks	56	Sensitivity	0.8947
					Specificity	0.9125
Wegner et al. (66)	2023	ARVA	Assessment of aortic diameter	50	MAD	2.4 mm/1.6 mm
Adam et al. (67)	2021	ARVA	Diameter measurement AA maximum	551	MAD	1.2 mm

AA, aortic aneurysm; ARVA, augmented radiology for vascular aneurysm; AUC, area under the curve; ICC, intraclass correlation coefficient; MAD, median absolute difference.

## 3 Frontiers of AI research in aortic CTA

### 3.1 Generative AI and image synthesis: from GANs to diffusion models

The clinical use of iodine contrast agents is often restricted due to patient conditions such as renal insufficiency or a history of allergic reactions (68). To overcome this limitation, the application of Artificial Intelligence to generate “virtual” CTA (Syn-CTA) from non-contrast CT (NCCT) has become a major research focus. The technological evolution in this domain shows a clear transition from the classic Generative Adversarial Networks (GANs) toward diffusion models.

Early efforts in image synthesis primarily relied on GANs. Chandrashekar et al. (69) compared Conditional-GAN and Cycle-GAN for synthetic aorta aneurysm tasks, finding that Cycle-GAN performed better in lumen segmentation and thrombus quantification. This is technically advantageous as Cycle-GAN utilizes a cycle-consistency loss, allowing it to be trained on unpaired NCCT-CTA data, thereby significantly reducing the dependence on strictly paired datasets. To overcome the inherent limitations of standard GANs in synthesizing fine pathological structures, such as intimal tears, researchers shifted towards more sophisticated Cascaded Architectures. Yin et al. (70) introduced a multi-stage cascaded GAN, which progressively refines the image by integrating residual attention blocks and a dual-attention mechanism, leading to significantly enhanced continuity of intimal tears. Similarly, Lyu et al. (71) designed a “generator-corrector-discriminator” tripartite architecture, where the “corrector” component is specifically trained to repair artifacts and discontinuities in the initial generated image, often outperforming traditional Pix2PixHD models in peak signal-to-noise ratio (PSNR) and structural similarity index measure (SSIM) metrics.

However, the inherent training instability and risk of generating artifacts associated with GANs have prompted the field to explore diffusion models. Unlike the adversarial game in GANs, the diffusion model architecture is based on a probabilistic denoising process, training a neural network to learn the reverse process of recovering a clear image from Gaussian noise (72, 73). This architecture theoretically ensures more stable training convergence than GANs.

These two generative paradigms exhibit distinct performance trade-offs. While cascaded GANs have been validated for their efficacy in high-speed, faithful domain transfer, diffusion models have demonstrated State-of-the-Art potential in generating high-fidelity, high-resolution textures. For Syn-CTA, Ullah et al. specifically employed a stable diffusion model to successfully synthesize CTA images depicting TBAD, demonstrating the capture of high-fidelity, pathology-specific features (74).

Inference efficiency is the primary bottleneck hindering the clinical translation of diffusion models. GANs require only a single forward pass for image generation, whereas diffusion models demand tens or even hundreds of iterative denoising steps (72), which can take minutes to generate a single CTA examination, potentially rendering them impractical for acute clinical settings. Although some GAN-generated images have achieved high diagnostic accuracy, the diagnostic reliability based on synthetic images still requires large-scale validation, suggesting a future research direction that may focus on combining the strengths of both—for instance, by utilizing GANs output as an initial inference step for a diffusion model to accelerate the sampling process.

### 3.2 Multi task learning: enhancing data efficiency and model synergy

In aortic CTA analysis, tasks such as segmentation, classification, and quantitative measurement are anatomically interdependent. Multi task learning (MTL) leverages this relationship by enabling a single model to learn multiple objectives simultaneously, thereby improving overall performance. Typical MTL architectures rely on either hard sharing or soft sharing encoder backbones.

Xiong et al. (75) introduced a representative hard sharing framework in which a shared 3D nnU-Net backbone extracts deep features from non-contrast CT images and feeds them into task specific heads responsible for CTA synthesis, true and false lumen segmentation, and aortic dissection detection. In contrast, the method proposed by Tan et al. (76) emphasizes stronger cooperation among tasks. Their 3D U Net jointly predicts landmark heatmaps, vessel segmentation maps, and direction fields, with each task offering complementary information: segmentation provides topological priors, direction fields impose geometric constraints, and a global optimization step during post processing further reduces anatomical inconsistencies. The advantages of MTL arise from parameter efficiency and an inherent regularization effect, as forcing the model to learn shared representations across tasks often mitigates overfitting and leads to superior performance compared with independently trained single task models. However, MTL remains challenged by negative transfer. When tasks are weakly related or pursue conflicting objectives, shared representations may degrade performance across all tasks. Balancing heterogeneous losses, such as Dice loss for segmentation and cross entropy for classification, continues to be a central methodological difficulty in MTL research.

### 3.3 Weak supervision: a pragmatic approach to annotation scarcity

Fully supervised learning relies on pixel-level “gold-standard” annotations manually delineated by experts. However, in aortic CTA, obtaining such high-fidelity labels is extremely time-consuming and expensive (77); a single detailed dissection annotation may require several hours. This imposes a substantial barrier to the development of large-scale models. Weakly supervised learning (WSL) provides a critical solution by leveraging low-cost and easily accessible “weak” labels, such as incomplete, inexact, and inaccurate supervision, to train models, thereby striking a balance between annotation efficiency and model performance (78, 79). In vascular imaging, WSL methods center on designing mechanisms capable of inferring complete 3D segmentation maps from inherently incomplete supervisory signals.

Given the difficulty of producing 3D annotations, a prominent direction is to utilize more attainable 2D labels. Guo et al. (80) proposed a framework validated on a multi-center CTA dataset including the aorta. Their architecture exploits easily annotated 2D maximum-intensity-projection (MIP) images, generating 3D pseudo-labels through a 2D–3D feature-fusion network and iteratively refining them using confidence estimation and uncertainty modeling. Notably, this method improved annotation efficiency by 80%, while achieving a Dice score comparable to the fully supervised baseline. Other studies explore more general vascular WSL strategies with promising transferability to aortic segmentation. For example, Chen et al. (81)'s 3D shape-guided local discrimination model utilizes public 2D vascular datasets (non-aortic) and employs adversarial learning alongside semantic consistency clustering to guide 3D aortic segmentation. Similarly, Ma et al. (82) addressed the blurred vascular boundaries in non-contrast CT by incorporating the anatomical prior that vessels exhibit an “elliptical” appearance, thus generating Gaussian-shaped pseudo-labels to supervise segmentation.

The advantages of WSL are clear: it dramatically reduces annotation demands and enables training with large-scale data. Nevertheless, its performance ceiling is inherently constrained by the weak supervisory information, which represents an intrinsic information bottleneck. Models may adopt suboptimal or biased strategies, such as focusing only on major vessels while neglecting small branches or subtle lesions. As a result, their reliability and robustness generally remain inferior to fully supervised models, and performance in complex pathologies (e.g., intimal tears) still requires further validation.

### 3.4 ViTs: capturing global dependencies

CNNs and their derivatives, including architectures such as U-Net, construct representations through progressively stacked local convolutional filters. This inherently limits their capacity to model long range dependencies. ViTs, originally developed in natural language processing, offer an alternative by enabling more effective global contextual modeling. CNNs requires substantial depth before their upper layers acquire an effectively global receptive field, whereas a ViTs establishes global interactions from its earliest stage. This capability is particularly relevant for aortic imaging. The aorta is an elongated and anatomically complex structure, and pathological processes such as type A aortic dissection may extend continuously from the ascending aorta to the abdominal segment. The global modeling capacity of ViTs is therefore theoretically advantageous for capturing long range morphological patterns and understanding the full extent of disease involvement.

Despite these strengths, fully transformer-based architectures still show variable performance in medical image segmentation. Hybrid designs that combine convolutional and transformer components, such as UNet Transformer (UNETR) (83) and Swin UNETR (84), have proven to be more reliable. These architectures integrate the strong local feature extraction of convolutional networks with the global contextual reasoning of transformers, demonstrating high diagnostic performance in vascular imaging tasks, such as achieving an AUC of 0.62 in predicting EVAR complications with 100% sensitivity, and 98.1% accuracy in aortic dissection detection with a processing time under 10 s (85, 86).

Its primary technical limitations lie in data requirements and computational burden. Vision Transformers lack the inductive bias inherent to convolutional networks, particularly the assumption of local spatial coherence, and therefore require substantially larger pretraining datasets to achieve performance comparable to convolutional models. In addition, the computational cost of self- attention grows quadratically with the length of the input sequence, creating significant challenges when processing high-resolution 3D CTA data, where the number of tokens becomes extremely large (87).

### 3.5 Foundation models: paving the way for generalist medical AI

The current research paradigm for aortic AI mainly focuses on specialized models optimized for specific diseases and single tasks. These methods typically utilize classic CNNs backbone networks, such as the early VGG-16 and ResNet-18 (88, 89). Although these backbone networks themselves are general-purpose, when applied to specific aortic tasks, the entire model architecture and training process are optimized for only a single target. While this single-task optimization strategy achieves high precision in specific applications, it also exposes inherent bottlenecks of low data utilization efficiency and poor cross-center generalization.

Foundation Models represent a potential path toward overcoming these limitations and advancing generalist medical AI. Their core concept involves self-supervised pre-training of a high-capacity general architecture such as large ViTs or hierarchical Transformers like the Swin-Transformer on a massive and diverse unlabeled medical imaging dataset to learn broad domain-agnostic visual feature representations (90, 91). This pre-training stage forces the model to extract foundational knowledge effective across multiple aortic pathological features. Subsequently the model can be efficiently fine-tuned with only a small amount of labeled data to simultaneously adapt to various downstream tasks ranging from the accurate segmentation of the aortic lumen and intraluminal thrombus to the prediction and classification of complex pathological changes. This “pre-train-and-fine-tune” paradigm theoretically increases data efficiency and model robustness against rare diseases substantially compared to traditional single-task methods. However, its technical and ethical challenges are also pronounced. Training a Foundation Model necessitates extremely high computational resources and unprecedented data volumes which pose severe challenges to data privacy protection and inter-institutional collaboration (91).

Furthermore, Foundation Models may inherit and amplify underlying systemic biases present in the training data introducing unpredictable safety risks into complex clinical decisions all of which constitute critical hurdles that must be addressed before their widespread clinical translation.

## 4 Challenges and perspectives of AI in aortic CTA

### 4.1 Data scarcity and generalizability barriers

Data scarcity remains a pervasive barrier to the development of robust medical AI models. The acquisition of imaging data is severely constrained by concerns regarding privacy, ethical hurdles, and disparities in imaging protocols across institutions, which creates isolated “data silos” (92). AI models designed for aortic CTA are no exception, as their training necessitates large-scale, high-quality datasets.

Current research on aortic CTA imaging predominantly focuses on single-center retrospective cohort studies. To ensure model training stability, the data curation procedures often apply strict exclusion criteria, such as excluding images with motion artifacts or bad bolus timing (56, 60). While the preprocessing steps yields highly pure datasets, it fails to mirror the complex, variable clinical scenarios encountered in emergency or trauma centers, thereby limiting model generalizability. The diversity in reconstruction methodologies significantly impacts AI performance. Specifically, Traditional FBP remains a baseline, but it yields specific noise textures and spatial resolutions (25). Modern techniques like IR and DLR are designed to enhance image quality. However, these different methodologies create “domain shifts”. AI models trained on FBP data may exhibit a significant performance drop when applied to DLR-enhanced images. Furthermore, variations in CT scanners pose major challenges. Different scanners utilize varied hardware and acquisition parameters, such as tube voltage and tube current. These settings directly alter the noise levels and Hounsfield Unit values within the aorta. For instance, lower tube voltage enhances contrast signals but increases image noise. Such physical discrepancies between vendors compromise the robustness of AI approaches during cross-platform validation.

Furthermore, the value of AI lies in long-term prognosis, such as monitoring aneurysm sac regression and the evolution of endoleaks. However, constructing datasets that track aortic morphology across multiple time points, including preoperative, postoperative, and long-term follow-up stages, is significantly more challenging than acquiring single-time-point diagnostic scans. Consequently, future research must move beyond simple cross-sectional datasets to establish 4D longitudinal registries capable of capturing the hemodynamic evolution of the aorta.

### 4.2 Annotation consistency and ground truth fidelity

In addition to data scarcity, the morphological complexity of aortic pathologies imposes unique constraints on the generation of ground truth labels. Unlike focal lesions such as lung nodules or solid tumors that typically span only a few slices, aortic diseases, specifically dissection, often involve extensive anatomical ranges and complex branch vessel involvement. This anatomical extent drastically increases the burden of annotation. Previous studies indicate that voxel-level manual annotation by an expert for a single case of Stanford Type B dissection requires, on average, more than 1 h (5). Furthermore, the ground truth used to train AI models typically relies on manual segmentation performed by radiologists with varying levels of experience. For instance, Huang et al. used annotations by one cardiovascular radiologist and one senior radiology resident (57).

Moreover, pathological complexity exacerbates inter-observer variability. As the intimal flap in a dissection is often extremely thin, tortuous, and spiral, delineating the precise boundaries between the true lumen and false lumen is highly subjective, particularly when the false lumen exhibits partial thrombosis or suboptimal contrast filling. To circumvent this difficulty, most current studies limit annotation to the contours of the true lumen and false lumen, omitting separate annotation of the intimal flap itself (43, 47). However, this simplification has significant limitations. Ignoring the intimal flap results in the loss of critical morphological information. This hinders the model's ability to learn the location of entry tears or the dynamic characteristics of the intima, thereby limiting clinical utility for precise surgical planning. Conversely, relying on a single annotator presents a different risk. While the individual's consistency might be high, the trained model may replicate that specific doctor's bias rather than anatomical reality.

To ensure the reliability of AI algorithms, future research must prioritize standardized multi-expert consensus annotations, specifically targeting these ambiguous anatomical regions.

### 4.3 Interpretability and clinical trust

The complex and non-transparent nature of AI decision-making, commonly referred to as the “black box” problem, restricts researchers’ ability to interpret the internal learning mechanisms and decision logic (93). While interpretability methods such as heatmaps and shapley additive explanations values are widely utilized in medical imaging research, the clinical demand for interpretability in aortic CTA extends beyond generic feature visualization.

For instance, although AI models developed by Pradella et al. (62) and Artzner et al. (61) have demonstrated high consistency with manual readings in diameter measurement, a solitary numerical output is clinically insufficient to establish surgical indications. To establish clinical trust, AI must present visual evidence supporting its decision logic in the future. Specifically, surgeons must be able to verify the exact cross-sections and axes selected by the models to ensure compliance with clinical guidelines. This verification is critical because a measurement deviation of even a few millimeters can determine the necessity for surgical intervention.

Prognostic assessment in aortic surgery, such as after EVAR, likewise requires a comparable level of interpretability. Although tools like PRAEVAorta 2, evaluated by Olivier et al. (64), and deep learning models by Talebi et al. (63) show potential in quantifying aneurysm sac regression and detecting endoleaks, a binary prediction of endoleak presence is insufficient to guide clinical decision-making. Surgeons require explicit visualization of the flow channel origin and the connection between the stent graft and the aneurysm sac to formulate effective re-intervention strategies.

Consequently, future aortic AI systems must evolve from generic interpretability toward geometric interpretability. This evolution is essential to bridge the gap between algorithmic accuracy and the rigorous safety standards required for aortic interventions.

## 5 Conclusion

This review comprehensively analyzes the evolution of AI in aortic CTA. Established applications have demonstrated robust clinical utility, where AI enhances image reconstruction, automates segmentation, and improves diagnostic and prognostic precision for conditions like aortic dissection and aneurysms. These advancements solidify CNN-based methods as a mature foundation for current practice. Building upon this foundation, we highlight a critical paradigm shift toward advanced frontiers. To address the limitations of traditional CNNs, the field is adopting next-generation architectures. Diffusion Models are establishing new standards for high-fidelity image synthesis, while ViTs capture essential global geometric dependencies. Furthermore, we posit that foundation models and self-supervised learning represent the definitive future, offering a path to overcome labeled data scarcity and achieve cross-center generalizability. Future research must now focus on curating large-scale, multi-center benchmark datasets and addressing interpretability challenges to fully realize the potential of generalist medical AI in aortic care.
